# Left Atrial Mitral Valve Chordae Which Disturbed the Mitral Leaflet Motion and Induced Mitral Regurgitation

**DOI:** 10.5761/atcs.cr.25-00030

**Published:** 2025-03-04

**Authors:** Toru Kameda, Tomohiro Mizuno, Kota Kawada, Tsubasa Yoshikawa, Koichi Sugiyama, Yuzo Katayama, Takeshiro Fujii

**Affiliations:** Department of Cardiovascular Surgery, Toho University Omori Medical Center, Tokyo, Japan

**Keywords:** mitral valve, cardiac anomaly, valve repair, mitral regurgitation, mitral chordae

## Abstract

Left atrial mitral valve chorda (LAMVC) is a rare congenital cardiac anomaly. The abnormal tissue band, like a mitral valve chorda, is attached to the left atrial wall on one side and mostly to the mitral valve leaflet on the other side and the band sometimes disturbs the mitral leaflet motion, followed by mitral regurgitation (MR). We encountered a case with a LAMVC which originated from a papillary muscle and attached to the posterior mitral annulus over the posterior leaflet and caused MR due to restricted mitral leaflet motion.

## Abbreviations

LAMVC
: left atrial mitral valve chorda
MR
: mitral regurgitation
TEE
: transesophageal echocardiography
AFMR
: atrial functional mitral regurgitation
TR
: tricuspid regurgitation

## Introduction

Left atrial mitral valve chorda (LAMVC) is a rare congenital cardiac anomaly. The abnormal tissue band, like a mitral valve chorda, is attached to the left atrial wall on one side and mostly to the mitral valve leaflet on the other side and the band sometimes disturbs the mitral leaflet motion, followed by mitral regurgitation (MR).^1)^ Pizzuti et al.^2)^ reported an incidence of 0.02% among approximately 30000 unselected echocardiogram cases. We encountered a case with a LAMVC which caused MR due to restricted mitral leaflet motion.

## Case Report

**Fig. 1:**
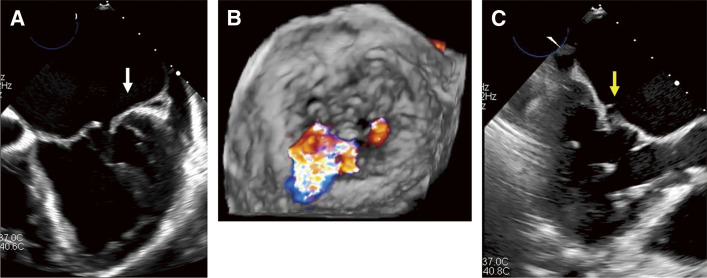
Preoperative transesophageal echocardiographic images. (**A**) Movement of the posterior mitral leaflet was restricted (white arrow). (**B**) A surgeon’s view. The mitral regurgitant jet came from the anterolateral P2 area. (**C**) An abnormal tissue appeared and disappeared on the posterior mitral leaflet along with the heart beating (yellow arrow).

A 76-year-old woman was admitted to our hospital with progressive dyspnea. She had chronic atrial fibrillation in her history. Her transesophageal echocardiography (TEE) showed severe atrial functional MR (AFMR) and tricuspid regurgitation (TR) with restricted movement of the lateral P2 leaflets, followed by MR (**Figs. 1A** and **1B**). We could not recognize an abnormal tissue on the mitral valve at this point.

**Fig. 2:**
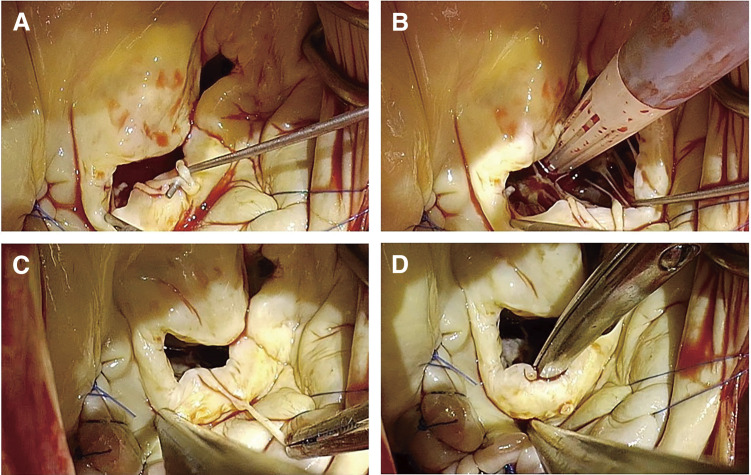
Intraoperative photographs of left atrial mitral valve chorda. (**A**) An abnormal band ran over the posterior mitral leaflet attached to the posterior mitral leaflet near the annulus and split into 2 parts. (**B**) One part was attached to the edge of the posterior mitral leaflet. (**C**) The other part was attached to a papillary muscle. (**D**) The posterior mitral leaflet after resection of the band.

The mitral valve was exposed via median sternotomy. In the operative field, an abnormal band ran over the posterior mitral leaflet (**Video 1**). The band was attached to the posterior mitral leaflet near the annulus on one side and split into 2 parts (**Fig. 2A**). One part was attached to the edge of the posterior leaflet, and the other one to a small papillary muscle on the other side (**Figs. 2B**–**2D**). It was speculated that the band pushed down the anterolateral P2 in the systolic phase and restricted leaflet movement, which might have worsened AFMR. The LAMVC was cut off and a pair of artificial chordae was placed at the anterior mitral leaflet, and a rigid ring was placed for annuloplasty. MR was reduced to trivial, and the movement of the posterior mitral leaflet improved. Postoperatively, after looking at the preoperative TEE images again, we recognized that an abnormal tissue appeared and disappeared along with the heartbeat (**Fig. 1C**). This was the LAMVC.

## Discussion

LAMVC is a rare congenital anomalous band, which looks like a mitral valve chorda. The band is attached to the left atrial wall on one side and mostly to the mitral valve leaflet on the other side.^1)^ Pizzuti et al.^2)^ reported an incidence of 0.02% among approximately 30000 unselected echocardiogram cases. The first report of LAMVC in 1947 described a band that bridged from the left atrial wall to the left atrial wall above the mitral valve and did not disturb the mitral valve function.^3)^ Reports of LAMVC have gradually increased with the progression of diagnostic technologies, and it has been recognized that the abnormal band sometimes causes MR, and surgical treatment is required.^4)^

In the case we described here, we did not recognize a LAMVC using TEE preoperatively, probably because the chorda was very slim and ran very close to the mitral valve. In addition, this case is exceedingly rare given that the chorda originated from the papillary muscle and ran over the posterior mitral leaflet to the posterior mitral annulus. The anomalous band disturbs coaptation in the systolic phase, resulting in a worsening AFMR.

Shirwaiker et al.^4)^ reported that tissue attached to the mitral leaflets is sometimes misdiagnosed as vegetation of endocarditis. If the anomaly is thoroughly understood, we can prevent misdiagnosis and unnecessary surgery.

## Conclusions

We reported a case of LAMVC which was the cause of severe MR. A LAMVC is very rare, but the opportunity to detect the disease is increasing with the widespread of TEE. LAMVC should be more understood to prevent misdiagnosis and unnecessary surgery.

## Declarations

### Ethical approval

The Institutional Review Board approved the study protocol and publication of data on August 10, 2024 (IRB approval number: M24078).

### Consent for publication

The patient provided informed written consent for the publication of the study data.

### Funding

This work was not supported by any grants or funding.

### Data availability statement

The data that support the findings of this study are available from the corresponding author upon reasonable request.

### Author contributions

TK and TM performed clinical work, drafted the manuscript, and designed the figures. KK, KS, and TY collected data. TM, YK, and TF were involved in planning and supervising the work. All authors have read and agreed to the published version of the manuscript.

### Disclosure statement

None declared.

## Supporting Information

Supplementary video Intraoperative video of the mitral valve.
